# Identification of *Toxocara* spp. eggs in dog hair and associated risk factors

**DOI:** 10.14202/vetworld.2017.798-802

**Published:** 2017-07-20

**Authors:** Tania O. Rojas, Camilo Romero, Rafael Heredia, Linda G. Bautista, Galia Sheinberg

**Affiliations:** 1 Department of Veterinary Medicine, Faculty of Agricultural Sciences and Natural Resources, University Center UAEM Amecameca, Autonomous University of Mexico State, Mexico; 2 Department of Veterinary Medicine, Research Academician of Animal Health, University Center UAEM Amecameca, Autonomous University of Mexico State, Mexico; 3 Department of Dermatology, Veterinary Center Mexico, Mexico City, Mexico

**Keywords:** dog hair, public health, risk factors, *Toxocara* eggs

## Abstract

**Aim:**

The aim of the study was to identify the presence of eggs of *Toxocara*spp. in dog hair and to identify any risk factors associated with this.

**Materials and Methods:**

A total of 96 dogs were sampled collecting hair from the head, perianal and hindquarters. Epidemiologic data from each animal were recorded to identify risk factors. The samples of hair were washed with solutions of distilled water, phosphate-buffered saline and Tween 20 detergent. Microscopic analysis was subsequently performed for the identification of eggs.

**Results:**

Out of the total dogs, 41.7% were positive for the presence of parasite egg in their hair. *Toxocara* eggs were found in hair from the head (14.5%), tail (20.8%), and limbs (10.4%). Dogs, younger than 12 months old, showed higher values (4.7%) of egg presence in the perianal area (p<0.05). The principal risk factors for the presence of eggs in hair were not deworming (odds ratio [OR]=3.60, p<0.004) and not brushing (OR=2.26, p<0.12).

**Conclusion:**

These results show that in the state of Mexico there is a high percentage of dogs contaminated with *Toxocara* spp. eggs in their hair. This should be seriously considered due to the potential problems of toxocariasis and the risk to public health.

## Introduction

*Toxocara canis* is a nematode parasite commonly found in the intestines of dogs [1]. It can be excreted as eggs in feces, and under appropriate conditions, they become infective in the environment during a period of 2-5 weeks [2]. Moreover, the parasite has the potential to invade other paratenic hosts such as humans [3]. The dog is the most common companion animal of humans, and this close contact exposes humans to possible zoonotic diseases like toxocariasis, which can cause mild symptoms or severe manifestations (slight fever, recurrent vomiting, headache, and abdominal pain) and in rare cases can sometimes lead to death [4,5]. Infection by *Toxocara* spp. is initiated through the ingestion of embryonated eggs and once the larvae in the eggs hatch they can migrate to different organs, causing syndromes such as visceral larva migrans, characterized by hepatic, pulmonary compromise, anemia, and eosinophilia; ocular larva migrans, in which the effects of pathological toxocariasis on the host are restricted to the eye and optic nerve, causing a significant decreased visual acuity and even total loss of the same [2,5].

A number of different risk factors for toxocariasis have been described. Among these are bad hygiene habits, ingestion of undercooked meat, food preparation and pica and specifically geophagia [6-9]. An important factor that has not been fully addressed is infection through human contact with dog hair [10]. Amaral *et al*. [6], however, have described how dog hair contaminated with *T. canis* at different stages of development is a source of infection and furthermore, higher densities of *Toxocara* eggs than detected in the soil have also been reported in dog hair [11].

Most studies of risk factors have been conducted in humans [1,4,8,12]; few have been conducted in dogs. Moreover, studies of risk factors for the presence of *Toxocara* eggs in dog hair have not been conducted in México. Considering this, the objective of this study was to evaluate the presence of *Toxocara* spp. eggs in dog hair and to evaluate risk factors for the presence of the eggs.

## Materials and Methods

### Ethical approval

With the consent of the pet owners, 96 dogs were sampled in the Southeast region of the State of Mexico from March 2013 to October 2013. This non-invasive collection method did not present any threat to these dogs. The sampling procedure required no specific permissions for the Southeast region of the State of Mexico.

### Sample collection and questionnaires

From each of the 96 dogs, samples were collected by trichotomy, with three samples of hair from different anatomical regions: Head, perianal area, and hindquarters. This resulted in a total of 288 hair samples being obtained which could be used to identify the presence of eggs of *Toxocara* spp. The samples were kept in a polyethylene bag with a zipper, labeled with the corresponding data (dog number and anatomical region from which the sample was collected) and kept frozen until analyses. A survey was completed by each of the dog owners, including questions related to epidemiological history and risk factors: Dog data (height, age, and hair length), health conditions, health habits (worming, flea presence, frequency of bathing, and hair cut service), and living conditions of the animals (stray habits, living with other dogs, wearing clothes, brushing, and type of floor where they live).

### Egg recovery technique

Samples were processed using the modified method of Overgaauw *et al*. [13]. They were weighed using an analytical scale (Velab™ model ES.1000H) and those samples weighing <2.5 mg were excluded. The samples were next washed with vigorous shaking in the presence of 0.2 ml of Tween 20 (label CIVEQ) and 40 ml of distilled water (JT. Baker^®^). After 10 min, the floating hair was transferred to another tube for a second wash with 40 ml of phosphate-buffered saline. After 10 min, the hair was discarded. The first and second washes were centrifuged at 800 ×*g* for 10 min and then the supernatant was decanted until approximately 1 ml. Once the sediments were re-suspended, these were transferred into a single tube to mix them. This was centrifuged at 800 ×*g* for 10 min and 1 ml of the supernatant retained. The re-suspended sediment was moved to an Eppendorf tube for a last centrifugation at 800 ×*g* for 10 min and approximately 100 µl of the supernatant was kept. Finally, each sample processed was observed under an optical microscope at 40× magnification to search for eggs of *Toxocara* spp.

### Statistical analysis

The degree of contamination of dog hair with eggs of *Toxocara* spp. was expressed as a percentage for the anatomical region and also per gram of hair. The percentages were also compared using the Kruskal–Wallis test [14]. The information obtained in the survey (age, breed, gender, etc.) was used to identify risk factors for *Toxocara* eggs in hair by calculating the odds ratio (OR) [15].

## Results

Of the sampled dogs, 41.7% (n=40) contained eggs of *Toxocara* spp. in their hair. In total, 67 eggs (not embryonated) were recovered from dogs’ hair; 19 from head, 38 from perianal region, and 10 from limbs. The perianal region showed a higher percentage of positive tests (20.8%) compared with the head (14.6%) and limbs (10.4%), however, the anatomical region was not found to be a predisposing factor in the overall population (Table-1). The mean number of eggs per gram (EPG) of hair was 1.4 EPG. Among anatomical regions, the mean number of EPG of hair was similar (p>0.05), with 1.23 in the head, 1.90 in the perianal region, and 1.0 in the limbs. Eggs density (per gram of hair) was not different (p>0.05) by gender (female 1.28 and males 1.60) or by the size of the dog (small 1.0; medium 1.45; and large 1.5). When the dogs were grouped by age (Table-2), younger dogs showed a higher density of *Toxocara* eggs in their perianal region (p<0.05).

**Table-1:** Presence of *Toxocara* eggs according to anatomical region.

Region	Positive n=44 (%)	Negative n=244	OR	p
Head	14 (14.58)	82	0.12	0.0001
Perianal region	20 (20.83)	76	0.20	0.0001
Limbs	10 (10.42)	86	0.08	0.0001
Total	44	244		

OR=Odds ratio

**Table-2:** Number of *Toxocara* eggs by anatomical region according to age group.

Eggs/g hair				

Age group	Months	Head	Perianal region	Limbs
Less than 12 months	8.50	1.00	4.66	1.00
More than 12 months	62.15	1.55	1.41	1.00
Variation coefficient	77.74	44.66	129.93	0.00
Kruskal–Wallis test				
Chi-square value	18.92	2.82	0.20	0.00
Degree of freedom	1	1	1	1
p>Ji-square	<0.0001	0.0926	0.0469	1.0000

Regarding the possible risk factors associated with the presence of *Toxocara* spp. eggs, the size of dog, age or hair length was not significant (Table-3). Deworming of dogs was, however, a protective factor (OR=0.27) and not deworming was a considerable risk factor (OR=3.60). The presence of fleas, bathing frequency, and grooming activities were not significant risk factors (Table-3).

**Table-3:** Evaluation of risk factors associated with the presence of *Toxocara* eggs in dog hair.

Variable	Positive n=40 (%)	Negative n=56	OR	p
Size				
Small-sized breed (<10 kg)	0 (0.0)	3	0.18	0.27
Medium-sized breed (10.1-25 kg)	36 (37.50)	47	1.72	0.39
Large-sized breed (>25 kg)	4 (4.17)	6	0.92	0.91
Age				
Young	12 (12.50)	18	0.90	0.82
Adult	19 (19.79)	28	0.90	0.80
Geriatric	9 (9.38)	10	1.35	0.57
Hair				
Short (≤0.5 cm)	15 (15.63)	18	1.66	0.25
Long (>0.5 cm)	25 (26.04)	38	0.78	0.58
Deworming				
Yes	12 (12.50)	34	0.27	0.003
No	28 (29.17)	22	3.60	0.0004
Presence of fleas				
Yes	21 (21.88)	26	1.27	0.55
No	19 (19.79)	30	0.78	0.55
Bathing frequency				
Frequent, every 1-4 months	19 (19.79)	32	0.67	0.35
Infrequent, less than once every 4 months	9 (9.38)	10	1.33	0.57
Never	12 (12.50)	14	1.28	0.50
Grooming frequency				
Frequent, every 1-4 months	15 (15.63)	23	0.86	0.72
Infrequent, less than once every 4 months	10 (10.42)	10	1.53	0.74
Stray habits				
Stray dog	15 (15.63)	19	1.16	0.71
Not a stray dog	25 (26.04)	37	0.85	0.71
Contact with other dogs				
Yes	28 (29.17)	39	1.01	0.91
No	12 (12.50)	17	0.98	0.97
Dressing habits				
Dressed	11 (11.46)	17	0.87	0.76
Not dressed	29 (30.21)	39	1.14	0.76
Brushed				
Yes	6 (6.25)	16	0.44	0.12
No	34 (35.42)	40	2.26	0.12
Floor where the dogs live				
Concrete	13 (13.54)	21	0.80	0.35
Grass	2 (2.08)	3	0.92	0.90
Mixed	20 (20.83)	24	1.33	0.48

OR=Odds ratio

In regard to the stray habits, dressing, and the type of floor, these were not risk factors. However, there was a tendency (p=0.12) to indicate that not brushing could be a risk factor (OR=2.26) or brushing a protective factor (OR=0.44) (Table-3).

## Discussion

The presence of *Toxocara* spp. in dogs represents a risk for public health due to the repercussions such as zoonotic diseases [5]. The results obtained in this study indicate that beside reports of *Toxocara* spp. eggs being recovered from feces and in public places, their presence in the hair of canines is a potential risk factor for the transmission of this parasite to other animals and humans. Furthermore, this data are supported by other studies; for example, Amaral *et al*. [6] and Öge *et al*. [16] also found that contamination of dog hair with *T. canis* at different stages of development represented a potential source of infection for humans.

Overgaauw *et al*. [13] analyzed 148 domestic dogs aged between 0.5 and 13 years of age, collecting hair samples from the lumbar region and flanks, however, they found eggs in 18 dogs (12.2%), which contrasts with our data presented here (40 positives, 41.7%). Both in the reported by Overgaauw *et al*. [13], as in this study embryonated eggs were not detected, however, Keegan *et al*. [3] reported that *Toxocara* eggs have the potential to become infective on dog which should not be ignored [17]. In another study, Keegan *et al*. [3] investigated 182 dogs, with 65 younger than a year and 117 older than a year, and hair samples were taken from the head, neck, back, and perianal region; 16 (8.8%) dogs were positive for *Toxocara* eggs. Again, the percentage of positive dogs here is lower than that in our present study.

Amaral *et al*. [6] analyzed hair from 100 stray dogs and found that 67% of dogs were positive for *T. canis* eggs. Of these, 95% were puppies, which indicates that stray dogs, and especially puppies, carry the eggs in their hair at higher densities than that reported from on the soil or in the environment [6,18]. Nevertheless, according to Overgaauw *et al*. [13] and Keegan *et al*. [3] a higher percentage of positive animals were found in adult dogs, similar to the results of this study, where 9 out of 10 geriatric dogs were positive for *Toxocara* spp. in the hair. Previous investigations of Keegan and Holland [3] and Overgaauw *et al*. [13] found similar results to those obtained in this study, indicating that age is an important factor for the presence of eggs in dog hair, associated with the *Toxocara* spp. biological cycle and the condition of the animal.

El-Tras *et al*. [19] compared the hair of 56 domestic dogs and 64 stray dogs: 6 domestic dogs (10.7%) and 17 stray dogs (26.6%) were positive for *Toxocara*. Although stray habits were not detected as a significant risk factor, numerically, hair from dogs with stray habits was more contaminated (26.0%) than hair from dogs without these habits (15.6%), which could be associated with the better care provided by the owner and a permanent home.

As with the mean number of EPG of hair reported by Overgaauw *et al*. (3.8 EPG) [13] and Keegan *et al*. (0.1 EPG) [3], the value found in this study (1.4 EPG) is considerably lower than the value obtained by Roddie *et al*. [11] (584 EPG), which is higher than that reported for soil. These differences may be explained by the fact that Roddie *et al*. [11] only used samples from stray dogs where the lack of attention and hygiene likely influenced in the high number of eggs in hair compared with the number found in animals with an owner. It is also possible that there is a relationship between the degree of contamination in the soil and in the hair. In places where the soil has a density of 0.0016-1.1 EPG [20-22], low concentrations of eggs in the hair were found, as in this study. This is important because soil has been considered as the principal source of infestation of *Toxocara* spp. to humans [23].

## Conclusions

A significant proportion of the dogs sampled in the Southeast region in the State of Mexico was contaminated with eggs of *Toxocara* spp. in their hair. The main risk factor for egg contamination was a lack of deworming, and the foremost protective measure against egg contamination was performing deworming. Although other factors were not found to be statistically significant risk factors, brushing dogs, avoiding stray habits, and grooming dogs at least every 4 months may contribute to the reduction of further contamination and infections.

## Authors’ Contributions

TOR and CR performed the experiments and prepared manuscript. LGB, GS, and RH interpreted the data and participated in draft and revision of the manuscript. All authors read and approved the final manuscript.
